# Whole exome sequencing for the identification of CYP3A7 variants associated with tacrolimus concentrations in kidney transplant patients

**DOI:** 10.1038/s41598-018-36085-w

**Published:** 2018-12-24

**Authors:** Minji Sohn, Myeong Gyu Kim, Nayoung Han, In-Wha Kim, Jungsoo Gim, Sang-Il Min, Eun Young Song, Yon Su Kim, Hun Soon Jung, Young Kee Shin, Jongwon Ha, Jung Mi Oh

**Affiliations:** 1 College of Pharmacy and Research Institute of Pharmaceutical Sciences, Seoul National University, Seoul, Republic of Korea; 2 Graduate school of clinical pharmacy, CHA University, Gyeonggi-do, Republic of Korea; 3 Department of Biomedical Science, Chosun University, Gwangju, Republic of Korea; 4 Department of Surgery, Seoul National University Hospital, Seoul, Republic of Korea; 5 Department of Laboratory Medicine, Seoul National University Hospital, Seoul, Republic of Korea; 6 Kidney Research Institute and Department of Medical Science, Seoul National University College of Medicine, Seoul, Republic of Korea; 7 R&D center, ABION, Inc., Seoul, Republic of Korea; 8 Molecular Medicine and Biopharmaceutical Sciences, Graduate School of Convergence Science and Technology, Seoul National University, Seoul, 08826 Republic of Korea

**Keywords:** Pharmacogenomics, Next-generation sequencing, Predictive markers

## Abstract

The purpose of this study was to identify genotypes associated with dose-adjusted tacrolimus trough concentrations (C_0_/D) in kidney transplant recipients using whole-exome sequencing (WES). This study included 147 patients administered tacrolimus, including seventy-five patients in the discovery set and seventy-two patients in the replication set. The patient genomes in the discovery set were sequenced using WES. Also, known tacrolimus pharmacokinetics-related intron variants were genotyped. Tacrolimus C_0_/D was log-transformed. Sixteen variants were identified including novel *CYP3A7* rs12360 and rs10211 by ANOVA. *CYP3A7* rs2257401 was found to be the most significant variant among the periods by ANOVA. Seven variants including *CYP3A7* rs2257401, rs12360, and rs10211 were analyzed by SNaPshot in the replication set and the effects on tacrolimus C_0_/D were verified. A linear mixed model (LMM) was further performed to account for the effects of the variants and clinical factors. The combined set LMM showed that only *CYP3A7* rs2257401 was associated with tacrolimus C_0_/D after adjusting for patient age, albumin, and creatinine. The *CYP3A7* rs2257401 genotype variant showed a significant difference on the tacrolimus C_0_/D in those expressing *CYP3A5*, showing its own effect. The results suggest that *CYP3A7* rs2257401 may serve as a significant genetic marker for tacrolimus pharmacokinetics in kidney transplantation.

## Introduction

Tacrolimus is an immunosuppressant drug widely used in most organ transplants^1^. Tacrolimus has successfully decreased the rejection rate and improved the outcome of many transplants^2^. However, individualization of tacrolimus therapy remains a challenge owing to the wide range of inter-individual variations in its pharmacokinetics (PK) and its narrow therapeutic index^3^. Accordingly, therapeutic drug monitoring (TDM) of tacrolimus leads to better clinical outcomes to prevent adverse effects and decrease the risk of allograft rejection in clinical settings.

Various factors have been reported to affect tacrolimus PK, such as concomitant drugs, genotypes, diet, and clinical values^4,5^. Notably, the *CYP3A5* rs776746 single-nucleotide polymorphism (SNP) found in intron 3 (6986A>G, CYP3A5*3 allele) is known to play a major role in the expression of the CYP3A5 enzyme via abnormal mRNA splicin^6^, which influences tacrolimus absorption and metabolism. Several guidelines have been implemented to compensate for this; for example, the Clinical Pharmacogenetics Implementation Consortium guidelines recommend a 1.5‒2-fold higher starting dose for CYP3A5 expressers, such as CYP3A5*1 carriers, compared to CYP3A5 non-expressers, such as CYP3A5*3 carriers^7^. Nevertheless, efforts are being made to discover the genetic determinants of tacrolimus PK because CYP3A5*3 is believed to account for 40‒50% of variations in tacrolimus dosage requirements^8^, and the correct dosage is still undetermined for a large proportion of patients. Recently, several other variants in *CYP3A4*^9^, *ABCB1*^10^, *POR*^11^, *NR1I2*^12^, and *SUMO4*^13^ genes have been found to affect tacrolimus PK. However, these genetic variants do not explain the substantial variability observed for tacrolimus PK.

Genome-wide screening studies constitute a potential new approach to identify novel SNPs. Next‒generation sequencing (NGS) has proven highly successful in identifying novel pathological genotypes^14,15^. Hence, this new technology is expected to reveal novel genetic variants to successfully predict tacrolimus PK. Additionally, a patient’s clinical condition can affect tacrolimus PK^2^; hence, clinical factors or laboratory variables associated with tacrolimus PK are required to predict the tacrolimus trough whole-blood concentration (C_0_). Therefore, this study aimed to identify genotypes via NGS and clinical factors associated with tacrolimus C_0_ after kidney transplantation (KT).

## Results

### Patient characteristics in the discovery and replication sets

The baseline demographic characteristics are presented in Table 1. There were no significant differences in age (46.2 ± 13.0 vs. 48.0 ± 12.4 years old, *P* = 0.386), body weight (60.8 ± 11.8 vs. 60.3 ± 9.3 kg, *P* = 0.778), or sex (61.3% vs. 66.7% male patients, *P* = 0.759) between the discovery and replication sets. Drugs with potential effects on tacrolimus PK, such as CYP3A inhibitors or inducers, were not administered.

**Table 1:** Baseline demographic characteristics for included subjects.

	Discovery patients (N = 75)	Replication patients (N = 72)	*P* value
Age (years)	46.2 ± 13.0	48.0 ± 12.4	0.386
Male, N (%)	46 (61.3)	48 (66.7)	0.759
Body weight (kg)	60.8 ± 11.8	60.3 ± 9.3	0.778
Deceased donor, N (%)	44 (58.7)	41 (53.9)	0.354
**Origin of kidney disease, N (%)**			
Diabetes	19 (25.3)	13 (18.1)	0.112
Hypertension	6 (8.0)	10 (13.9)	0.147
Glomerulonephritis	26 (34.7)	26 (36.1)	0.603
PKD	7 (9.3)	7 (9.7)	0.512
Others	7 (9.3)	4 (5.6)	0.544
Unknown/CGN	10 (13.3)	12 (16.7)	0.470
**Clinical variables**			
Hematocrit (%)	34.8 ± 3.9	33.9 ± 3.6	0.114
Total cholesterol (mg/dL)	151.8 ± 36.1	152.3 ± 40.2	0.928
Albumin (g/dL)	3.5 ± 0.5	3.5 ± 0.6	0.995
Total bilirubin (mg/dL)	0.7 ± 0.3	0.7 ± 0.4	0.913
Alanine transaminase (U/L)	15.2 ± 10.2	16.3 ± 10.3	0.494

PKD, polycystic kidney disease; CGN, chronic glomerulonephritis; Data are presented as a number with percentage for categorical variables and a mean with standard deviation for continuous variables.

### Sequencing and alignment quality

After sequence mapping, the mean coverage depth was 57.78x (41.09–105.5), and 87.87% (77.87–96.09%) of target regions showed >20x coverage (Supplementary Table S1). After variant calling, 293,531 variants were identified and used for association. The data quality and quantity are described in Supplementary Table S1.

### Variants associated with tacrolimus trough concentrations using whole-exome and intron data

**Figure 1:**
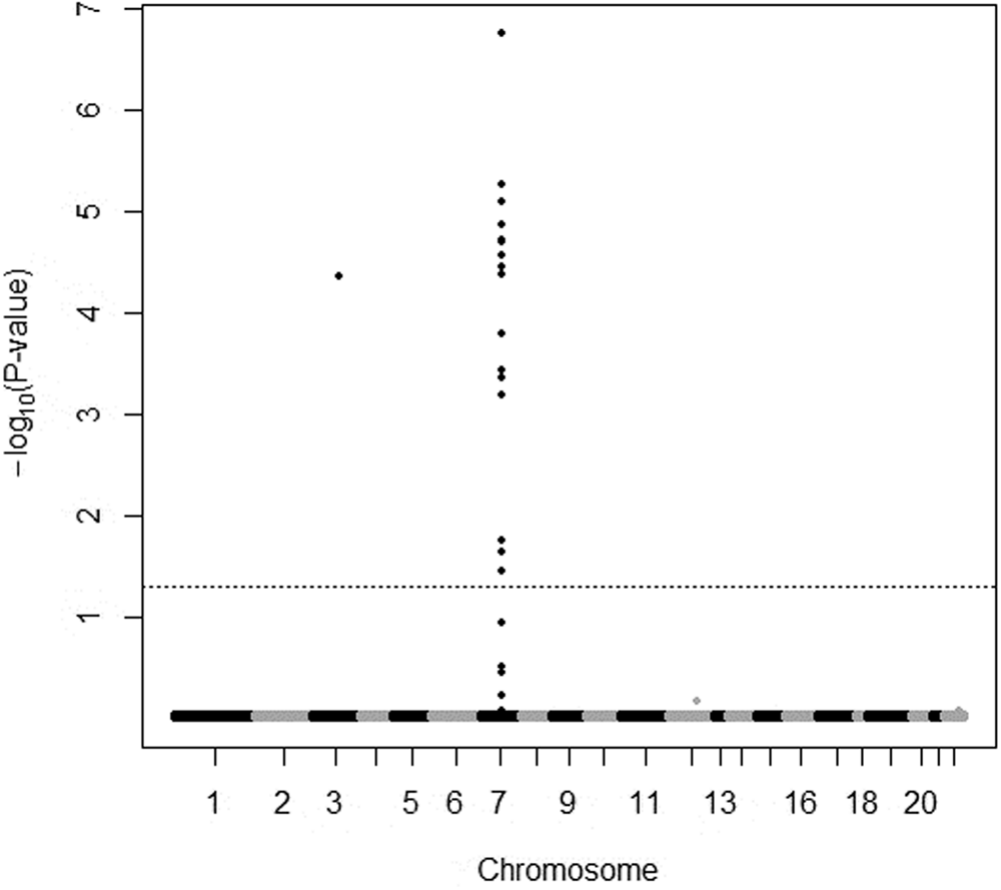
Manhattan plots of variants associated with dose-adjusted tacrolimus trough concentrations on day 7 after transplantation. The dotted horizontal line shows the cutoff of *P* = 0.05 after false discovery rate correction.

**Figure 2:**
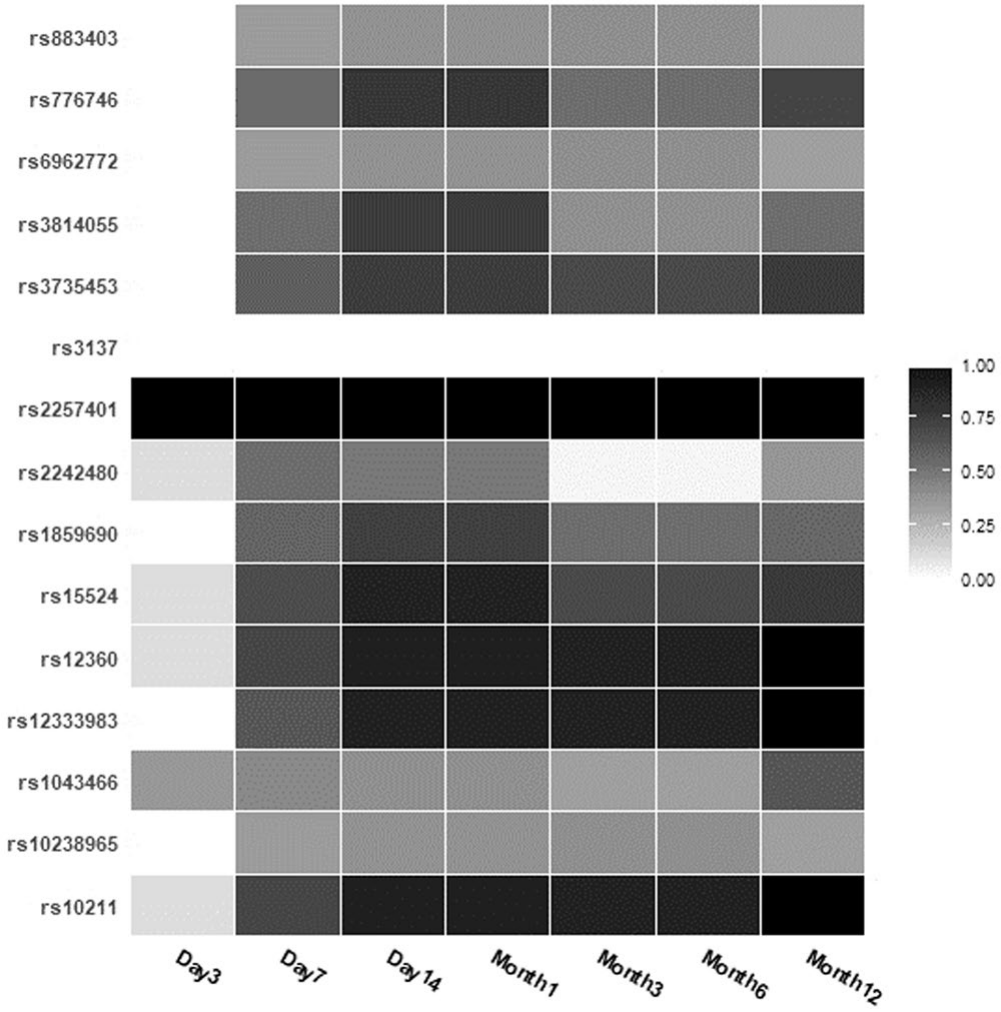
Heat map plots of variants commonly associated with dose-adjusted tacrolimus trough concentrations from day 3 to 1 year after transplantation. The color was scaled based on an adjusted *P* value of variants associated with tacrolimus trough levels (black, highly significant association; white, no significant association).

**Figure 3:**
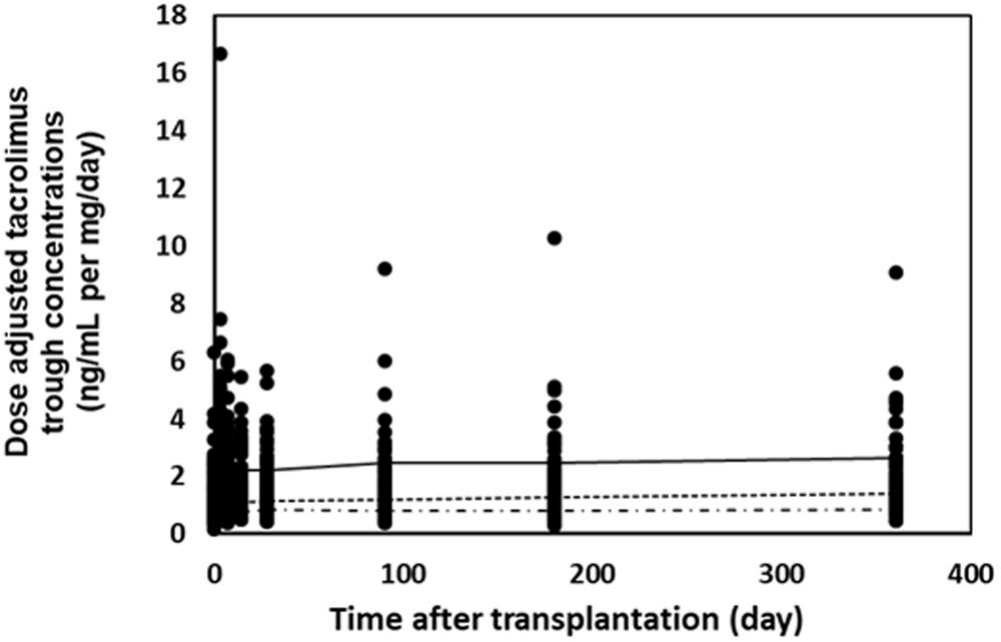
Dose-adjusted tacrolimus trough concentrations in patients after kidney transplantation. Solid, dotted, and dashed lines indicated the mean of dose-adjusted tacrolimus trough concentrations in carriers with *CYP3A7* rs2257401 GG, CG, and CC genotypes, respectively.

In total, 2,900 tacrolimus C_0_ measurements were collected from patients during the first year after transplantation in the discovery set. The median C_0_ per patient was 37 points, with a range from 28‒94 points. After analyzing the association between the variants and the tacrolimus dose-adjusted trough concentration (C_0_/D) using ANOVA, genes such as *NR1I2* on chromosome 3 and *PTCD1*, *CPSF4*, *ZNF789*, *ZKSCAN5*, *FAM200A*, *ZSCAN25*, *CYP3A5*, *CYP3A7*, and *CYP3A4* on chromosome 7 were strongly associated with tacrolimus C_0_/D (Fig. 1 and Supplementary Figure S1) at one or more time points. All the variants conformed with the Hardy-Weinberg Equilibrium (*P* > 0.001) and matched those reported for East Asian populations (Supplementary Table S2). Sixteen variants were significantly associated with tacrolimus C_0_/D after FDR (*P* < 0.05) from 3 days to 1 year after transplantation at each time point (Fig. 2 and Supplementary Table S3). The *CYP3A7* rs2257401 C to G substitution was the most significant variant for increasing the tacrolimus C_0_/D in all periods and was the only variant that strongly associated with tacrolimus C_0_/D on day 3 after transplantation (between *P* = 1.74 × 10^−7^ and *P* = 0.0138, Supplementary Table S3 and Fig. 3). The fourteen other variants associated with tacrolimus C_0_/D were found at multiple times between day 7 and 1 year (*CPSF1* rs883403 and rs1043466; *ZNF789* rs6962772; *FAM200A* rs10238965; *ZSCAN25* rs1859690 and rs3735453; *CYP3A5* rs15524 and rs776746; *CYP3A7* rs10211, rs12360, and rs2257401; *CYP3A4* rs12333983 and rs2242480; and *NR1I2* rs3814055). The mean tacrolimus C_0_ in relation to these seven variants in drug-metabolizing enzymes, including *CYP3A4*, *CYP3A5*, and *CYP3A7* and the nuclear receptor gene *NR1I2*, at different time points after transplantation are presented in Supplementary Table S4.

### Association of haplotypes with tacrolimus trough concentrations

**Figure 4:**
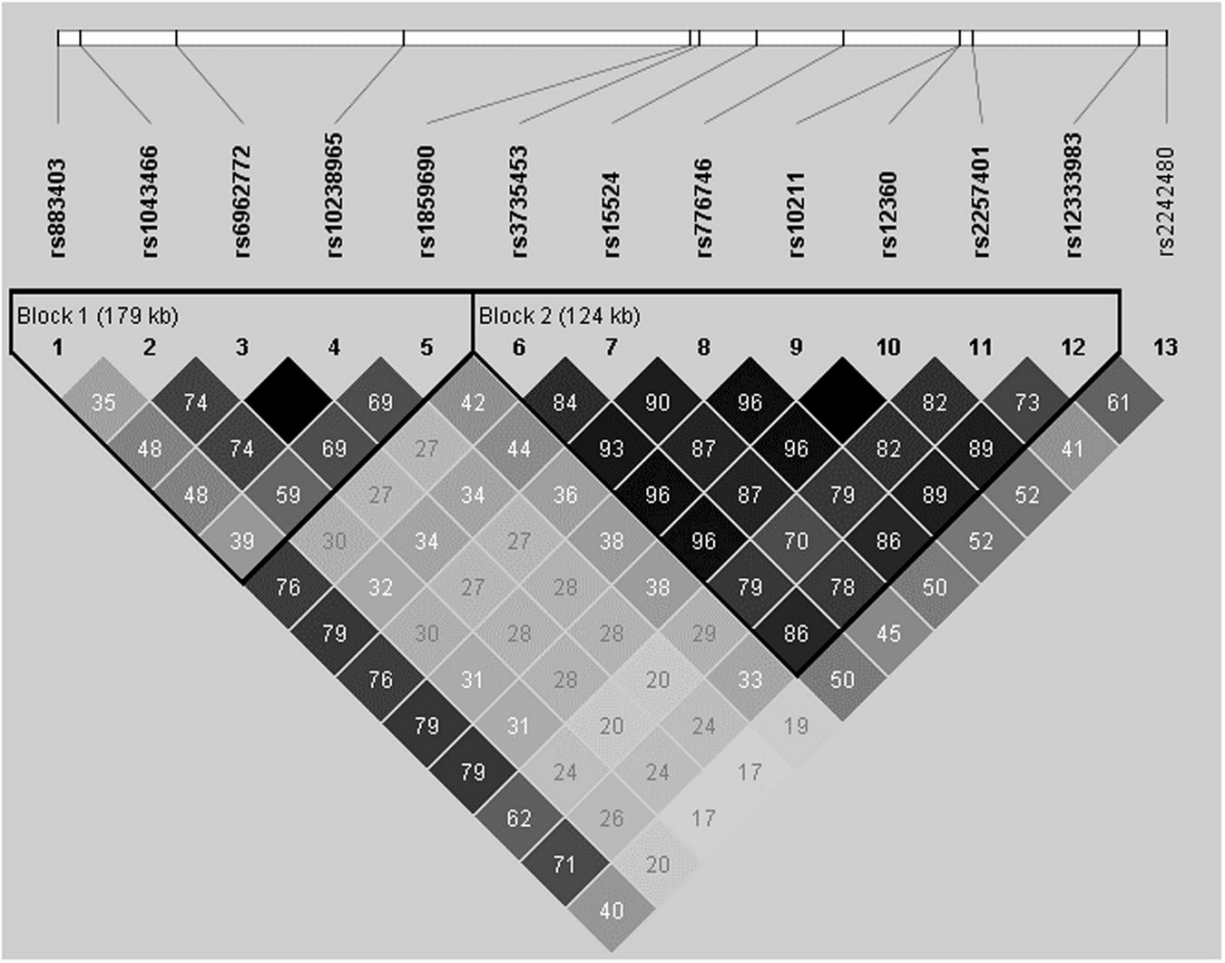
Haplotype plots of variants on chromosome 7 commonly associated with dose-adjusted tacrolimus trough concentrations from day 7 to 1 year after transplantation.

A haplotype distribution was constructed for thirteen variants in seven genes associated with tacrolimus C_0_/D, including *CPSF1*, *ZNF789*, *FAM200A*, *ZSCAN25*, *CYP3A5*, *CYP3A7*, and *CYP3A4* on chromosome 7. Linkage disequilibrium (LD) structures were designated by the *r*^2^ values shown in Fig. 4. One haplotype block consisted of *CPSF1* rs883403 and rs1043466, *ZNF789* rs6962772 and *FAM200A* rs10238965, and *ZSCAN25* rs1859690; the other consisted of *ZSCAN25* rs3735453 and *CYP3A5* rs15524 and rs776746; *CYP3A7* rs10211, rs12360, and rs2257401; and *CYP3A4* rs12333983. The *ZNF789* rs6962772, *FAM200A* rs10238965, and *CYP3A7* rs10211 and rs12360 SNPs displayed complete linkage; therefore, *ZNF789* rs6962772 and *CYP3A7* rs10211 were not used in further analyses. There was a high degree of LD between *CYP3A7* rs2257401 and *CYP3A5* rs776746 (*r*^2^ = 0.79) and a moderate degree of LD between *CYP3A4* rs2242480 and *CYP3A5* rs776746 (*r*^2^ = 0.50). The diplotype frequencies of *CYP3A5* rs15524 and rs776746, *CYP3A7* rs10211 and rs2257401, and *CYP3A4* rs12333983, including CAGCA-CAGCA, TGAGT-TGAGT, and others, are summarized in Supplementary Table S5. Haplotype combinations showed that the CAGCA-CAGCA, TGAGT-TGAGT, and other diplotypes were observed at frequencies of 9.3%, 45.3%, and 44.2%, respectively. The TGAGT-TGAGT diplotype was strongly associated with increased tacrolimus C_0_/D compared to CAGCA-CAGCA at different time points during the first year after transplantation (Supplementary Table S6).

### Association of genotypes and clinical factors with tacrolimus trough concentrations

Biochemical data including the hematocrit, serum albumin, total cholesterol, total bilirubin, alanine transaminase, and creatinine were measured during the study period (Supplementary Table S7). A mixed effect model analysis was performed to further identify genetic variants and clinical factors. Patient age at transplantation and serum creatinine were significantly positively associated with tacrolimus C_0_/D in the discovery set (*P* < 0.001, Table 2). The C–G substitution rate for *CYP3A7* rs2257401 and T–C substitution rate for *CYP3A4* rs2242480 were significantly increased in patients with a dose-adjusted tacrolimus C_0_/D after adjusting for age and serum creatinine levels (*P* < 0.001).

**Table 2:** Results from the final linear mixed model for tacrolimus dose-adjusted trough concentrations in kidney transplant recipients.

Variables	Univariate		Multivariate	
	Estimate	95% CI	Estimate	95% CI
**Discovery set (N** **=** **75)**				
Age	1.018	1.010 − 1.027	1.014	1.009−1.020
Serum creatinine	1.064	1.025–1.105	1.053	1.015–1.093
Serum albumin	0.942	0.890–0.997	0.959	0.906–1.015
*CYP3A5* rs776746 A>G	1.654	1.444–1.894	1.201	0.639–1.080
*CYP3A7* rs2257401 C>G	1.736	1.539–1.959	1.784	1.401–2.271
*CYP3A4* rs2242480 T>C	1.733	1.436–2.090	1.227	1.022–1.474
**Replication set (N** **=** **72)**				
Age	1.004	0.994–1.014		
Serum creatinine	1.066	1.031–1.102	1.055	1.020–1.091
Serum albumin	0.925	0.878–0.975	0.946	0.897–0.998
*CYP3A5* rs776746 A>G	1.867	1.634–2.133	2.071	1.464–2.931
*CYP3A7* rs2257401 C>G	1.740	1.495–2.026	0.886	0.634–1.237
*CYP3A4* rs2242480 T>C	1.552	1.299–1.854	1.024	0.858–1.221
**Combined set (N** **=** **147)**				
Age	1.011	1.004–1.018	1.010	1.005–1.015
Serum creatinine	1.066	1.039–1.093	1.057	1.030–1.084
Serum albumin	0.935	0.899–0.971	0.956	0.919–0.994
*CYP3A5* rs776746 A>G	1.750	1.591–1.925	1.245	0.993–1.561
*CYP3A7* rs2257401 C>G	1.727	1.568–1.902	1.328	1.074–1.642
*CYP3A4* rs2242480 T>C	1.634	1.854–1.441	1.134	0.993–1.294

### Validation of genotypes and clinical factors associated with tacrolimus trough concentrations in the replication set

In total, 2,940 tacrolimus C_0_ measurements were obtained from patients during the first year after transplantation in the replication set. The median C_0_ per patient was 34 points (ranging from 27‒77 points). Seven variants associated with tacrolimus C_0_/D in the discovery set, including *CYP3A5* rs15524 and rs776746; *CYP3A7* rs10211, rs12360, and rs2257401; and *CYP3A4* rs12333983 and rs2242480, were genotyped by SNaPshot assay in the replication set. The allele frequencies of these variants are presented in Supplementary Table S8 and were comparable with the frequencies in the discovery set. Mixed effect model analysis showed that serum albumin and creatinine were positively associated with tacrolimus C_0_/D in the replication set (*P* < 0.05, Table 2). After adjusting for serum albumin and creatinine, only the A to G substitution rate of *CYP3A5* rs776746 was significantly increased in patients with dose-adjusted tacrolimus C0/D (*P* < 0.001). The mean tacrolimus C0/D based on the *CYP3A5* rs776746 genotypes at different time points are summarized in Supplementary Table S9.

### Genotypes and clinical factors associated with tacrolimus trough concentrations in the combined set

A combined analysis of both the discovery and replication sets showed that only the *CYP3A7* rs2257401 variant was associated with tacrolimus C_0_/D after adjusting for patient age, serum albumin, and creatinine (*P* < 0.05, Table 2). Next, the effect of the *CYP3A7* rs2257401 and *CYP3A5* rs776746 variants on the tacrolimus C_0_/D in patients expressing *CYP3A5* (CYP3A5*1/*1 or CYP3A5*1/*3) and not expressing *CYP3A5* (CYP3A5*3/*3) was examined. There was a significant difference in tacrolimus C_0_/D in carriers of the *CYP3A7* rs2257401 genotype for those expressing CYP3A5 (*P* < 0.001), but no significant difference in tacrolimus C_0_/D was found in carriers of the *CYP3A7* rs2257401 genotype for those not expressing *CYP3A5* (*P* = 0.180, Table 3). The *CYP3A7* rs2257401 CC genotype was not found in patients not expressing *CYP3A5*.

**Table 3:** Tacrolimus dose-adjusted trough concentrations in *CYP3A7* rs2257401 genotypes classified by different patients expressing *CYP3A5*.

CYP3A5	rs2257401	N	Daily dose adjusted tacrolimus trough concentrations (ng/mL per mg/day)						
			Day 3	Day 7	Day 14	Month 1	Month 3	Month 6	Month 12
Expressers*	GG or CG	52	1.61 ± 0.99	1.23 ± 0.75	1.05 ± 0.43	1.15 ± 0.69	1.20 ± 0.78	1.17 ± 0.65	1.33 ± 0.66
	CC	15	0.99 ± 0.46	0.70 ± 0.35	0.74 ± 0.26	0.82 ± 0.35	0.78 ± 0.28	0.74 ± 0.25	0.82 ± 0.25
Nonexpressers	GG	71	3.26 ± 2.29	2.58 ± 1.39	2.17 ± 1.09	2.09 ± 0.97	2.25 ± 1.23	2.18 ± 1.33	2.37 ± 1.36
	CG	9	2.11 ± 1.61	1.80 ± 1.23	1.83 ± 1.05	2.04 ± 1.29	1.84 ± 0.92	2.00 ± 0.87	2.16 ± 1.29

Data are presented as a mean with standard deviation. *P < 0.05 analyzed by a mixed effect model after adjusted by patient age, serum albumin, and creatinine. *CYP3A5* expressers are carriers with CYP3A5*1/*1 or CYP3A5*1/*3.

## Discussion

To our knowledge, this is the first study to perform a combined whole-exon and intron association analysis with clinical factors associated with tacrolimus C_0_/D in Korean KT recipients. Sixteen variants including *CYP3A* family genes with novel SNPs, *CYP3A7* rs12350 and rs10211, were identified to affect tacrolimus C_0_/D. The *CYP3A7* rs2257401 SNP variant was most significantly associated with tacrolimus C_0_/D and related after adjusting for patient age, serum albumin, and creatinine levels.

CYP3A7 is a major CYP enzyme in fetal livers. However, it is rapidly downregulated within the first few years postpartum^16^. Sim *et al*. reported that CYP3A7 protein expression is higher than CYP3A5 expression in 10% of adult livers, which may be relevant for the metabolism of various substrates^17^. The underlying mechanism is assumed to involve a C–G substitution in *CYP3A7* rs2257401, which causes a Thr/Arg amino acid substitution, thereby decreasing enzyme activity in an *in vitro* study using human HEK293 cells^18^. However, few studies have questioned the role of the CYP3A7 enzyme. Recombinant CYP3A7 supersomes showed lower metabolic activity than CYP3A4 and CYP3A5 supersomes^19^. The metabolic velocity of CYP3A7 for tacrolimus was lower than that of CYP3A5, whereas the K_m_ value of CYP3A7 was higher than that of CYP3A5 in a baculovirus-expressed CYP system^20^.

For calcineurin inhibitors, a few studies have described the effect of CYP3A7*1C, a promoter variant affecting their metabolism; however, their results were inconclusive^21–23^. Carriers of the CYP3A7*1C allele maintained a higher level of expression into adulthood and required higher doses of cyclosporine^21,22^. Nonetheless, Elens *et al*. reported that this polymorphism had no effect on tacrolimus C_0_/D and dose requirements in liver transplant recipients^23^. *CYP3A7* rs2257401 has been reportedly associated with the area under the concentration-time curve of tacrolimus in healthy Korean subjects^12^. *CYP3A7* rs12360 and rs10211 are SNPs in distance of 100 bp in the 3′-UTR region. Few microRNAs are reported to bind with rs12360 suggesting to modify gene expression, whereas none are reported to bind rs10211^24^.

The *CYP3A7* rs2257401 allele frequency was demonstrated to differ substantially between ethnic groups. The minor allele frequency (MAF) of the rs2257401 C allele was different in Tanzanian (MAF = 0.62), Chinese (MAF = 0.28), Saudi Arabian (MAF = 0.17), and Caucasian (MAF = 0.08) populations^18^. The MAF of the *CYP3A7* rs2257401 genotype in the present study was 30.7%, which was similar to findings from a previous study on healthy Korean subjects^21–23,25^. *CYP3A7* rs2257401 variation was reported to be higher in individuals of Asian descent; this information showed that this genotype might be a useful biomarker, as it was the major significant variant associated with tacrolimus C_0_/D in the Korean population.

The present results showed that patient age was associated with an increase in the tacrolimus C_0_/D, which was consistent with previous findings^8,26^. Additionally, serum albumin levels of patients were positively associated with an increase in the tacrolimus C_0_/D. A previous study reported the influence of albumin levels on tacrolimus clearance^27^. Serum albumin levels in the present study were increased soon after transplantation; however, they returned to baseline within 1 month. Restoration of albumin levels could increase the levels of bound tacrolimus in the blood and thus reduce tacrolimus clearance. Furthermore, an apparent correlation between serum creatinine levels and tacrolimus C_0_/D was previously observed^28^, which was consistent with the present findings. Thus, any increase in systemic exposure to tacrolimus would increase serum creatinine levels^29^.

In the linear mixed model (LMM), the variants selected from each genotype and clinical values were used as a covariate. All were associated with tacrolimus C_0_/D for each; however, only one variant was retained in the final model. The final LMM included *CYP3A7* rs2257401 to best explain tacrolimus C_0_/D in the discovery set (*P* < 0.05 using a *chi-square* test between the model with rs2257401 and the model with rs776746). *CYP3A5* rs776746 was selected in the final LMM in the replication set (non-significant *chi-square* test between two models; which indicates that model fitness’s are similar). Such results suggested that even though the final LMM in discovery set was not confirmed, the role of *CYP3A7* rs2257401 was still important. The difference of the results may have occurred because *CYP3A5* rs776746 variants were detected more frequently in the replication set. This explanation coincides with previous reports that the *CYP3A7* is strongly but not completely linked to *CYP3A5* rs776746^12,30^, which means a larger study is required to verify the importance of *CYP3A7* rs2257401 for personalized tacrolimus therapy.

A high degree of LD between *CYP3A7* rs2257401 and *CYP3A5* rs776746 (*r*^2^ = 0.79) was observed and reported in Birdwell *et al*., an another tacrolimus PK study without the effect of *CYP3A7* rs2257401^31^. To determine the sole effect of CYP3A7 in the present study, the recipients were categorized as those characterized by *CYP3A5* expression and those lacking *CYP3A5* expression, and the effect of *CYP3A7* variants on tacrolimus C_0_/D was examined in each group. A substantial difference was observed in the C_0_/D between patients with and without the *CYP3A7* rs2257401 genotype among those characterized by *CYP3A5* expression, which suggests its own effect on the CYP3A7 enzyme.

In our study of the discovery set, the CYP3A4*1G (rs2242480, 20239T>C) variant was found to have a positive association with tacrolimus C_0_/D, which was consistent with previous reports^12^. The *CYP3A4* rs2242480 SNP located on intron 10 has been reported to be associated with tacrolimus C_0_/D in Asian KT recipients, but the functional role of this polymorphism remains unclear^32,33^. CYP3A4*1B (rs2740574, A>G) has been reported to be associated with tacrolimus C_0_/D^34^; however, it was not tested in the current study because no variant was reported in individuals of Asian descent by dbSNP^35^.

Although numerous studies have attempted to investigate whether three *ABCB1* polymorphisms, 1236C>T (rs1128503), 2677G>A/T (rs2032582), and 3435C>T (rs1045642), affect tacrolimus PK, few have successfully shown any association^10,27^. In the present study, no associations were observed between tacrolimus C_0_/D and these *ABCB1* polymorphisms, which was identical with Hesselink *et al*.^34^. Although the absorption of tacrolimus is affected by P-glycoprotein, the roles of these *ABCB1* polymorphisms remain unclear.

Except *CYP3A* family, other gene variants were also identified. When Bonferroni correction was applied, all the variants except *NR1I3* rs3814055 were also significant (threshold of *P*-value < 2.5 × 10^–6^, data not shown)^36^. *PTCD1* encodes a mitochondrial protein and rs28495024 was related to distinct mtDNA gene expression in Cohen *et al*.^37^. *CPSF4* rs883403 and rs14043466 have been identified in genome studies on various diseases. However, too many unrelated diseases have been reported, and the exact role of the variant is in question^38^. *ZKSCAN5* is suggested to be involved in transcriptional regulation and rs3137 is reported to bind with microRNAs, resulting in a modified function^39^. The role of *ZNF789* gene variant has not been reported in any database. For *ZSCAN25*, which is also known as *ZNF498*, a study suggests that its gene region overlaps with *CYP3A5*, but no evidence was presented^40^. Owing to no known functions on the pharmacokinetics of the genes, the genes were not selected for verification in the replication set.

Even though sequencing methods have advanced, NGS is still challenging. Numerous studies have compared sequencers and bioinformatics tools, but the best technique has not yet been concluded^41,42^. In our data, while the average coverage was larger than 55, the fact that 13% of the exome is covered with less than 20x suggests that it would be challenging to call these regions. The percent bases in the target reads was low, but it was similar with previous studies; nonetheless, improvements are still required^43,44^.

To our knowledge, the statistical tools to test the power of NGS even with continuous variables, such as drug concentrations rather than disease risk, are not established^45^. When roughly estimated by the Genome Power Calculator^46^, the power to detect *CYP3A7* rs2257401 was supposedly over 95%, though it was still low for detecting rare variants. The current research was designed to identify clinically practical genotypes and we expected the results to be meaningful. As the sample size of this study was considered not large enough to identify mutations, those were excluded for validation.

Although the present study clearly identified the relationship between genotypes and tacrolimus trough levels, certain limitations exist. The exact function of *CYP3A7* was not determined; therefore, further studies are required to determine the physiological role of the *CYP3A7* enzyme in drug metabolism. Furthermore, future prospective studies are warranted to adequately characterize the effects of the *CYP3A7* variant and clinical factors on the tacrolimus PK.

In conclusion, the present study reported that *CYP3A7* polymorphisms are strongly associated with individual differences in the tacrolimus C_0_/D. Therefore, genotyping of the *CYP3A7* rs2257401 polymorphism may help optimize personalized tacrolimus dosages for KT recipients.

## Materials and Methods

### Study design and population

The current retrospective observational cohort study was performed in a single center at Seoul National University Hospital (SNUH). Patients aged 18 years or older who underwent KT from January 2007 to September 2014 and were treated with tacrolimus (Prograf, Astellas Pharma Korea, Inc., Seoul, Korea) during the first year after transplantation were included in this study. Patients were excluded if they underwent other organ transplants or if they received desensitization therapy due to the presence of donor-specific antibodies or ABO blood-type incompatibility. A total of 147 patients were included in this study; seventy-five were placed in the discovery set, and seventy-two were placed in the replication set. This study was approved by the ethics committee of SNUH (IRB No. C-1504-009-662) and was performed in accordance with the Guidelines for Good Clinical Practices and the Declaration of Helsinki^47^. Two written informed consent documents, for study participation and genetic testing of blood samples, were obtained from all study subjects. All subjects studied were of Korean ethnicity and no prisoners were included.

### Immunosuppressive regimen

Induction therapy included 20 mg preoperative intravenous basiliximab (Simulect, Novartis Pharmaceuticals, East Hanover, NJ, USA) and 4 days postoperative basiliximab or rabbit antithymocyte globulin (Thymoglobulin, Sanofi, Paris, France) for 4‒7 days, as well as 0.5 g preoperative intravenous methylprednisolone (Methysol, Alvogen Korea, Seoul, Korea). Subsequent maintenance triple immunosuppressive therapy was employed, including tacrolimus, mycophenolate (Cellcept, Roche, Nutley, NJ, USA; or Myfortic, Novartis, East Hanover, NJ, USA), and steroids. Tacrolimus was prescribed at an initial oral dose of 0.075 mg/kg twice daily from the day before transplantation and was adjusted thereafter according to TDM methods. The daily dose was adjusted to maintain the tacrolimus C_0_ at 10‒12 ng/mL during the first month after KT, 8‒10 ng/mL until 3 months, 6‒8 ng/mL until 6 months, and 4‒6 ng/mL thereafter. Prednisolone was gradually tapered to 5‒10 mg/day before the patients were discharged and 10 mg/day by 2 weeks after discharge. Additionally, 500 mg mycophenolate mofetil or 360 mg mycophenolate sodium was administered every 12 h on the day of transplantation; the dose was adjusted according to the side effects.

### Tacrolimus trough concentrations and clinical variable data collection

Ethylenediaminetetraacetic acid-anticoagulated whole blood samples were collected from kidney recipients visiting the department of Surgery just before the morning dose was administered. The tacrolimus C_0_ was measured at regular intervals for the first year after transplantation. The tacrolimus C_0_ was analyzed in whole blood by liquid-chromatography tandem mass spectrometry (LC-MS/MS) using a Waters 2795 Alliance HT system (Waters Ltd., Watford, UK) and a Quattro micro API tandem mass spectrometer (Micromass, Manchester, UK) as described previously^48^. The mean tacrolimus C_0_ values were calculated for the following periods after KT: days 1‒3, days 4‒7, days 8‒14, and days 15‒28, months 1‒3, months 4‒6, and months 7‒12. The clinical information for each patient, including the age at transplantation, sex, body weight, donor source, concomitant medications, and previous history of transplantation, were obtained; the biochemical laboratory variables, including the hematocrit, total serum cholesterol, total bilirubin, albumin, and creatinine, were collected at the time of tacrolimus C_0_ sampling at regular intervals for the first year after transplantation.

### Genomic DNA isolation and whole exome sequencing

Genomic DNA (gDNA) was isolated from recipient whole blood using the QuickGene DNA whole blood kit (Kurabo Industries, Osaka, Japan) according to the manufacturer’s instructions. The purity and concentration of gDNA was measured using a NanoDrop (Thermo Fisher Scientific, Grand Island, NY, USA). The exons and untranslated regions (UTRs) in the discovery set were genotyped by whole exome sequencing (WES). WES was conducted using 1 μg fragmented gDNA, which was captured using the Sure Select Human All Exon kit V5 + UTRs (Agilent Technologies, Santa Clara, CA, USA) and amplified. The libraries were evaluated for quality and quantified with a High Sensitivity DNA kit using a Bioanalyzer (Agilent Technologies). Enrichment was conducted using an Ion OneTouch ES (Thermo Fisher Scientific). Samples were loaded on two Ion PI chip Kits and sequenced on the Ion Proton System using an Ion PI Hi-Q Sequencing 200 Kit (200-bp read length, Thermo Fisher Scientific). Reads were mapped against the human reference genome (hg19) using the Torrent Mapping Alignment Program version 4.0.6 (Thermo Fisher Scientific). Variant calling was performed by running the Torrent Variant Caller plugin version 4.4.3.3 with the recommended optimized parameters for exome sequencing. Individual SNPs were excluded if they were monomorphic or had a low minor allele frequency (MAF; <1%). Variants were annotated and classified as deleterious by SNVrap (http://jjwanglab.org/snvrap)^49^.

### Sanger sequencing

Sanger sequencing confirmed all of the samples with the *CYP3A7* rs2257401 candidate variant (Supplementary Figure S2). Variant-rich regions were amplified by PCR (primer sets are listed in Supplementary Table S10). Purified PCR amplicons were directly sequenced with the BigDye Terminator v3.1 Cycle Sequencing Kit (Thermo Fisher Scientific) according to the manufacturer’s protocol. Subsequent analysis was performed using a 3130xl Genetic Analyzer (Thermo Fisher Scientific).

### SNaPshot or SNPtype assay

Non-targeted regions including intronic variants identified by the SureSelect kit in the discovery set were genotyped using the SNaPshot Multiple Kit (Thermo Fisher Scientific) or SNPtype assay (Fluidigm, San Francisco, CA, USA) according to the manufacturer’s instructions. The PubMed database was used to find previously reported variants within the intron related to tacrolimus PK. Variants with an MAF higher than 1% in East Asian populations were selected. A total of ten variants in six genes, including *CYP3A4*, *CYP3A5*, *ABCB1*, *NR1I2*, *SLCO1B3*, and *SUMO4*, were genotyped using the SNaPShot assay (Supplementary Table S11), while a total of thirteen variants in nine genes, including *ABCB1*, *ABCC2*, *ABCG2*, *CYP3A5*, *NR1I2*, *POR*, *PPARA*, *PPARD*, and *SUMO4*, were genotyped using the SNPtype assay (Supplementary Table S12). NGS sequencing results were confirmed by a SNaPshot assay for all of the samples at *CYP3A7* rs12360 and *CYP3A5* rs15524. Seven variants, chosen based on their potential gene functions, including *CYP3A7*, and associated with tacrolimus C_0_ in the discovery set were further confirmed by a SNaPshot assay in the replication set.

### Statistical analyses

The tacrolimus C_0_/D was calculated by dividing the tacrolimus C_0_ by the corresponding daily dose. The log-transformed tacrolimus C_0_/D was used for further analysis. Linear regression and analysis of variance (ANOVA) were used to test for the association between C_0_/D and each polymorphism for each time period according to additive models. After false discovery rate (FDR) correction, a value of *P* < 0.05 was considered statistically significant^50^. The Hardy-Weinberg equilibrium was tested for each SNP using the chi-square test to compare the observed and expected genotype frequencies. SNPs were excluded based on divergence from the Hardy-Weinberg equilibrium (*P* > 0.001). Continuous or categorical variables at the baseline characteristics between the discovery and replication sets were analyzed with Student’s *t*-test and the chi-square test or Fisher’s exact test, respectively. LMM analysis was performed to identify variants and clinical covariates associated with the tacrolimus C_0_/D at different time points in all discovery set, replication set, and combined set. An LMM was constructed to include genotypes and clinical covariates that affected the repeated measures of C_0_/D in a univariate analysis with a significance value of *P* < 0.20. Subsequently, a second LMM was performed with selected covariates with a significance level of *P* < 0.05 for all retrieved variables in the full model. Statistical analysis was performed using R software (version 3.3.2, www.r-project.org). Haplotypes and haplotype frequencies were calculated using the Haploview software (v4.2, Massachusetts Institute of Technology, Cambridge, MA, USA).

## Electronic supplementary material

Supporting Information
