# Author Correction: Whole exome sequencing for the identification of CYP3A7 variants associated with tacrolimus concentrations in kidney transplant patients

**DOI:** 10.1038/s41598-020-58225-x

**Published:** 2020-02-07

**Authors:** Minji Sohn, Myeong Gyu Kim, Nayoung Han, In-Wha Kim, Jungsoo Gim, Sang-Il Min, Eun Young Song, Yon Su Kim, Hun Soon Jung, Young Kee Shin, Jongwon Ha, Jung Mi Oh

**Affiliations:** 1 College of Pharmacy and Research Institute of Pharmaceutical Sciences, Seoul National University, Seoul, Republic of Korea; 2 Graduate school of clinical pharmacy, CHA University, Gyeonggi-do, Republic of Korea; 3 Department of Biomedical Science, Chosun University, Gwangju, Republic of Korea; 4 Department of Surgery, Seoul National University Hospital, Seoul, Republic of Korea; 5 Department of Laboratory Medicine, Seoul National University Hospital, Seoul, Republic of Korea; 6 Kidney Research Institute and Department of Medical Science, Seoul National University College of Medicine, Seoul, Republic of Korea; 7 R&D center, ABION, Inc., Seoul, Republic of Korea; 8 Molecular Medicine and Biopharmaceutical Sciences, Graduate School of Convergence Science and Technology, Seoul National University, Seoul, 08826 Republic of Korea

Correction to: *Scientific Reports* 10.1038/s41598-018-36085-w, published online 24 December 2018

The original version of this Article omitted an affiliation for Young Kee Shin. The correct affiliations for Young Kee Shin are listed below:

College of Pharmacy and Research Institute of Pharmaceutical Sciences, Seoul National University, Seoul, Republic of Korea.

Molecular Medicine and Biopharmaceutical Sciences, Graduate School of Convergence Science and Technology, Seoul National University, Seoul 08826, Republic of Korea.

This error has now been corrected in the PDF and HTML versions of the Article and in the accompanying Supporting Information file.

