# Supplementary material for: Whole exome sequencing for the identification of CYP3A7 variants associated with tacrolimus concentrations in kidney transplant patients
Source: Sci Rep. 2018 Dec 24;8:18064. doi: 10.1038/s41598-018-36085-w (PMC6305386; doi:10.1038/s41598-018-36085-w)
Supplement: Supplementary file 1 — Supporting Information [file 41598_2018_36085_MOESM1_ESM.pdf]

Supporting Information for

Whole exome sequencing for the identification of CYP3A7 variants associated with tacrolimus concentrations in kidney transplant patients

Minji Sohn[1], Myeong Gyu Kim[1,2], Nayoung Han[1], In-Wha Kim[1], Jungsoo Gim[3], Sang-Il Min[4], Eun Young Song[5], Yon Su Kim[6], Hun Soon Jung[7], Young Kee Shin[1,8], Jongwon Ha[4*], and Jung Mi Oh[1*]

[1]College of Pharmacy and Research Institute of Pharmaceutical Sciences, Seoul National University, Seoul, Republic of Korea

[2]Graduate School of Clinical Pharmacy, CHA University, Gyeonggi-do, Republic of Korea

[3]Department of Biomedical Science, Chosun University, Gwangju, Republic of Korea

[4]Department of Surgery, Seoul National University Hospital, Seoul, Republic of Korea

[5]Department of Laboratory Medicine, Seoul National University Hospital, Seoul, Republic of Korea

[6]Kidney Research Institute and Department of Medical Science, Seoul National University College of Medicine, Seoul, Republic of Korea

[7]R&D center, ABION, Inc., Seoul, Republic of Korea

[8]Department of Molecular Medicine and Biopharmaceutical Sciences, Graduate School of Convergence Science and Technology, Seoul National University, Seoul, Republic of Korea

Contents of this File

Supplementary Figure S1

Supplementary Figure S2

Supplementary Table S1

Supplementary Table S2

Supplementary Table S3

Supplementary Table S4

Supplementary Table S5

Supplementary Table S6

Supplementary Table S7

Supplementary Table S8

Supplementary Table S9

Supplementary Table S10

Supplementary Table S11

Supplementary Table S12

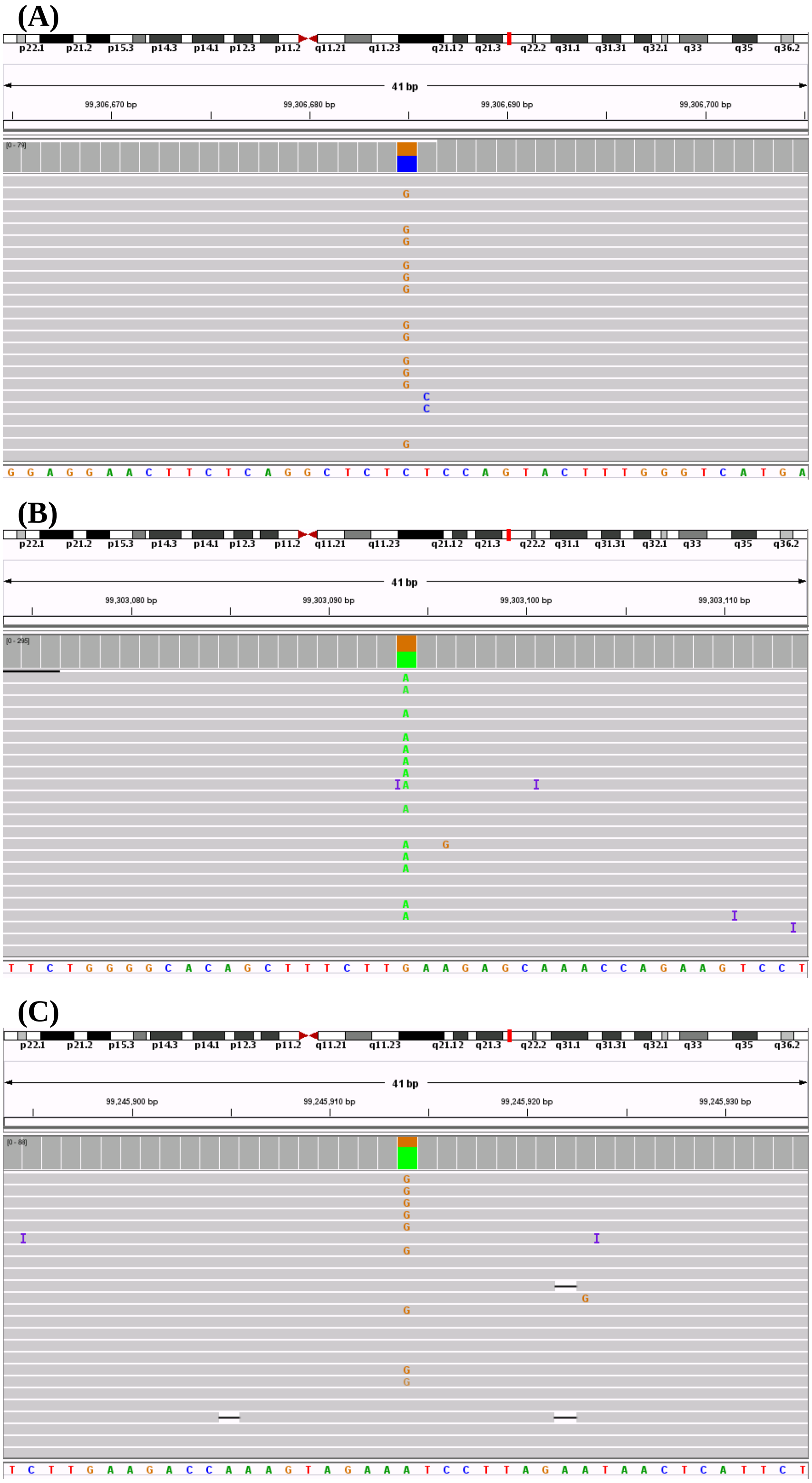

Supplementary Figure 1 Alignment of exome sequences showing (**A**) *CYP3A7* rs2257401 variant, (**B**) *CYP3A7* rs12360 variant, and (**C**) *CYP3A5* rs15524 variant by Integrative Genomics Viewers.

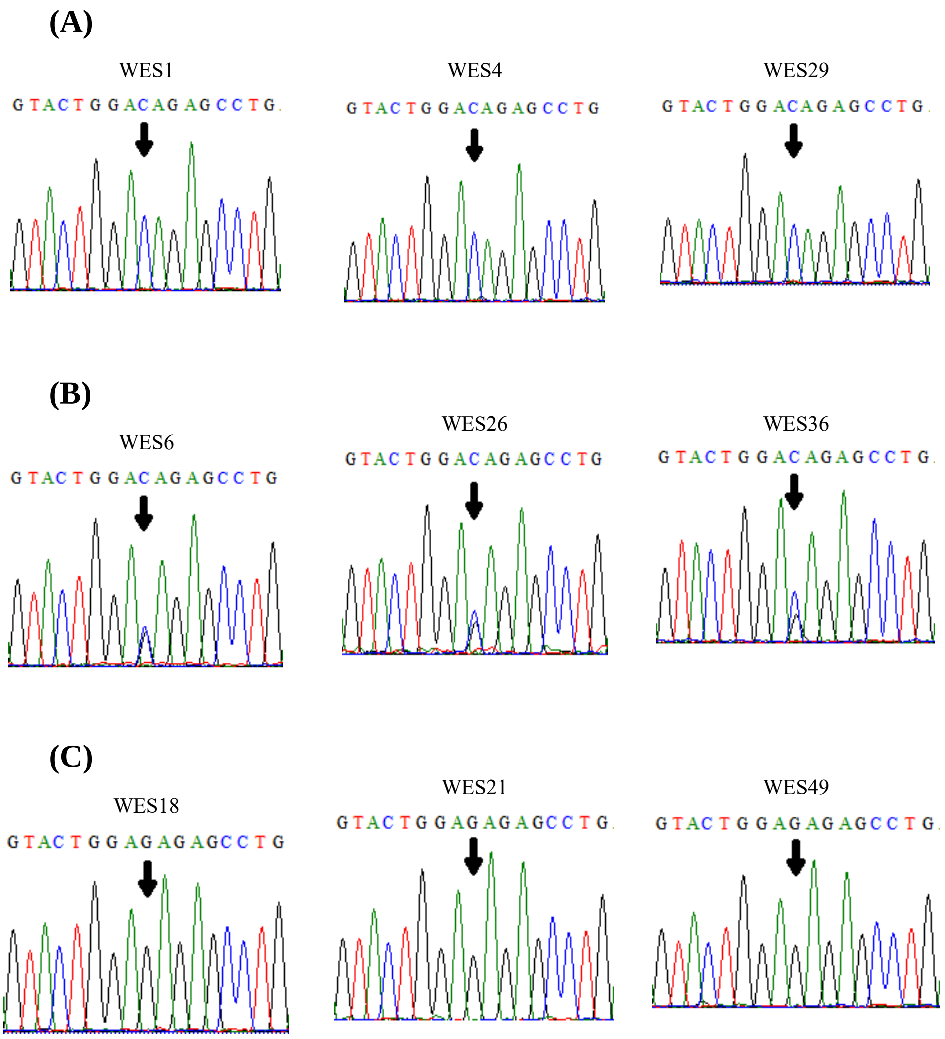

Supplementary Figure 2 Detection of *CYP3A7* rs2257401 polymorphisms, (**A**) CC variant, (**B**) CG variant, and (**C**) GG variant, by Sanger sequencing. The arrows in the electropherograms show the position of the variants

Supplementary Table S1 Summary of exome sequencing quality and exome variant metrics (N = 75)

Quality measure	Median (range)
Total reads (N)	6489589247 (4430895977-10858208267)
Base in target reads (N)	74569526
Number of mapped reads (N)	48194415 (31278758-93723614)
Percent base in target reads (%)	66.78 (47.69-72.28)
Average base coverage depth (x)	57.78 (41.09-105.5)
Uniformity of base coverage per target (%)	95.14 (94.38-95.8)
Captured regions with coverage > 20 (%)	87.87 (77.87-96.09)
Mean coverage of target region (x)	75.38 (53.11-79.74)
Homozygous variants (N)	256321 (253001-258746)
Heterozygous variants (N)	37211 (34785-40530)

Supplementary Table S2 Allele frequencies of variants associated with daily dose-adjusted tacrolimus trough levels in the discovery set

SNP	Position	Allele	Gene	Consequence	MAF	HWE P value
rs28495024	7:99016683	G>C	*PTCD1*	3'UTR	0.300	0.034
rs883403	7:99047978	A>G	*CPSF4*	Synonymous	0.313	0.228
rs1043466	7:99054289	T>G	*CPSF4*	3'UTR	0.273	0.020
rs6962772	7:99081730	A>G	*ZNF789*	5'UTR	0.313	0.228
rs3137	7:99130875	C>T	*ZKSCAN5*	3'UTR	0.280	0.119
rs10238965	7:99145845	C>T	*FAM200A*	Synonymous	0.313	0.228
rs1859690	7:99227172	T>C	*ZSCAN25*	Synonymous	0.213	0.230
rs3735453	7:99229435	A>G	*ZSCAN25*	3'UTR	0.273	0.245
rs15524	7:99245914	C>T	*CYP3A5*	3'UTR	0.293	0.230
rs776746	7:99270539	A>G	*CYP3A5*	Intron	0.273	0.245
rs10211	7:99302994	G>A	*CYP3A7*	3'UTR	0.267	0.181
rs12360	7:99303094	C>T	*CYP3A7*	3'UTR	0.267	0.181
rs2257401	7:99306685	C>G	*CYP3A7*	missence	0.307	0.397
rs12333983	7:99354114	A>T	*CYP3A4*	3'UTR	0.260	0.358
rs2242480	7:99361466	T>C	*CYP3A4*	Intron	0.207	0.863
rs3814055	3:119500035	T>C	*NR1I2*	5'UTR	0.233	0.737

MAF, minor allele frequency; HWE, Hardy-Weinberg equilibrium

Supplementary Table S3 Association of variants with daily dose-adjusted tacrolimus trough levels by the analysis of variance of linear regression in the discovery set (N=75)*

Variants	Day 3	Day 7	Day 14	Month 1	Month 3	Month 6	Month 12
rs28495024	1.38×10^{-01}	6.51×10^{-04}	5.42×10^{-02}	5.42×10^{-02}	7.18×10^{-04}	9.26×10^{-04}	9.26×10^{-04}
rs883403	1.38×10^{-01}	3.73×10^{-04}	1.03×10^{-03}	1.03×10^{-03}	3.37×10^{-04}	3.37×10^{-04}	4.93×10^{-02}
rs1043466	5.96×10^{-02}	1.58×10^{-04}	9.23×10^{-04}	9.23×10^{-04}	5.97×10^{-04}	5.97×10^{-04}	8.25×10^{-03}
rs6962772	1.38×10^{-01}	3.73×10^{-04}	1.03×10^{-03}	1.03×10^{-03}	3.37×10^{-04}	3.37×10^{-04}	4.93×10^{-02}
rs3137	1.38×10^{-01}	2.26×10^{-02}	1.28×10^{-01}	1.28×10^{-01}	1.18×10^{-02}	1.18×10^{-02}	3.56×10^{-01}
rs10238965	1.38×10^{-01}	3.73×10^{-04}	1.03×10^{-03}	1.03×10^{-03}	3.37×10^{-04}	3.37×10^{-04}	4.93×10^{-02}
rs1859690	1.38×10^{-01}	2.72×10^{-05}	1.39×10^{-05}	1.39×10^{-05}	1.03×10^{-04}	1.03×10^{-04}	1.29×10^{-02}
rs3735453	1.38×10^{-01}	1.99×10^{-05}	1.07×10^{-05}	1.07×10^{-05}	2.48×10^{-05}	2.48×10^{-05}	3.94×10^{-03}
rs15524	1.07×10^{-01}	7.94×10^{-06}	1.87×10^{-06}	1.87×10^{-06}	2.48×10^{-05}	2.48×10^{-05}	3.84×10^{-03}
rs776746	1.38×10^{-01}	3.51×10^{-05}	7.79×10^{-06}	7.79×10^{-06}	1.03×10^{-04}	1.03×10^{-04}	5.01×10^{-03}
rs10211	1.07×10^{-01}	5.46×10^{-06}	1.55×10^{-06}	1.55×10^{-06}	4.08×10^{-06}	4.08×10^{-06}	9.43×10^{-04}
rs12360	1.07×10^{-01}	5.46×10^{-06}	1.55×10^{-06}	1.55×10^{-06}	4.08×10^{-06}	4.08×10^{-06}	9.43×10^{-04}
rs2257401	1.38×10^{-02}	1.74×10^{-07}	4.90×10^{-07}	4.90×10^{-07}	1.53×10^{-06}	1.53×10^{-06}	9.39×10^{-04}
rs12333983	1.38×10^{-01}	1.34×10^{-05}	1.77×10^{-06}	1.77×10^{-06}	4.08×10^{-06}	4.08×10^{-06}	9.43×10^{-04}
rs2242480	1.07×10^{-01}	4.14×10^{-05}	2.94×10^{-04}	2.94×10^{-04}	9.18×10^{-03}	9.18×10^{-03}	3.87×10^{-02}
rs3814055	1.38×10^{-01}	4.37×10^{-02}	1.02×10^{-01}	1.02×10^{-01}	3.42×10^{-01}	3.42×10^{-01}	4.51×10^{-02}

**P* value was adjusted by a false discovery rate correction.

Supplementary Table S4 Tacrolimus trough levels over time according to genotypes in the discovery set (N=75)

SNP (*Gene*)	Allele	N	Daily dose adjusted tacrolimus trough levels (ng/mL per mg/day)						
			Day 3	Day 7	Day 14	Month 1	Month 3	Month 6	Month 12
rs28495024	GG	41	3.18±2.61	2.54±1.31*	1.96±0.90	2.31±1.08	2.31±1.52*	2.30±1.63*	2.46±1.53*
(*PTCD1*)	GC	23	2.06±1.42	1.43±1.02	1.35±0.98	1.44±0.85	1.25±0.60	1.31±0.77	1.52±0.97
	CC	11	1.24±0.56	0.95±0.56	1.03±0.81	1.06±0.62	0.97±0.51	1.02±0.58	1.07±0.55
rs883403	AA	38	3.20±2.65	2.57±1.32*	2.03±0.90*	2.36±1.09*	2.35±1.56*	2.36±1.68*	2.47±1.59*
(*CPSF4*)	AG	27	2.16±1.51	1.52±1.03	1.40±0.99	1.51±0.84	1.35±0.62	1.39±0.75	1.63±0.92
	GG	10	1.13±0.44	0.87±0.52	0.81±0.32	0.90±0.37	0.83±0.25	0.87±0.32	0.94±0.35
rs1043466	TT	44	3.26±2.55	2.52±1.36*	2.04±1.06*	2.33±1.13*	2.26±1.50*	2.29±1.62*	2.44±1.51*
(*CPSF4*)	TG	21	1.74±1.05	1.32±0.71	1.20±0.40	1.33±0.46	1.27±0.55	1.25±0.57	1.46±0.88
	GG	10	1.13±0.44	0.87±0.52	0.81±0.32	0.90±0.37	0.83±0.25	0.87±0.32	0.94±0.35
rs6962772	AA	38	3.20±2.65	2.57±1.32*	2.03±0.90*	2.36±1.09*	2.35±1.56*	2.36±1.68*	2.47±1.59*
(*ZNF789*)	AG	27	2.16±1.51	1.52±1.03	1.40±0.99	1.51±0.84	1.35±0.62	1.39±0.75	1.63±0.92
	GG	10	1.13±0.44	0.87±0.52	0.81±0.32	0.90±0.37	0.83±0.25	0.87±0.32	0.94±0.35
rs3137	CC	42	3.09±2.56	2.43±1.32	1.92±0.92	2.25±1.09	2.25±1.53	2.25±1.64	2.39±1.55
(*ZKSCAN5*)	CT	24	2.11±1.56	1.53±1.11	1.44±1.04	1.52±0.91	1.32±0.66	1.37±0.81	1.59±0.97
	TT	9	1.19±0.43	0.92±0.52	0.85±0.32	0.95±0.36	0.88±0.21	0.92±0.30	0.99±0.33
rs10238965	TT	38	3.20±2.65	2.57±1.32*	2.03±0.90*	2.36±1.09*	2.35±1.56*	2.36±1.68*	2.47±1.59*
(*FAM200A*)	TC	27	2.16±1.51	1.52±1.03	1.40±0.99	1.51±0.84	1.35±0.62	1.39±0.75	1.63±0.92

	CC	10	1.13±0.44	0.87±0.52	0.81±0.32	0.90±0.37	0.83±0.25	0.87±0.32	0.94±0.35
rs1859690	TT	40	3.24±2.68	2.59±1.37*	2.11±1.03*	2.39±1.13*	2.34±1.52*	2.36±1.65*	2.45±1.56*
(*ZSCAN25*)	TC	26	1.99±1.12	1.43±0.77	1.21±0.56	1.39±0.57	1.29±0.63	1.33±0.65	1.62±0.91
	CC	9	1.08±0.47	0.74±0.39	0.76±0.25	0.84±0.32	0.80±0.25	0.78±0.26	0.83±0.28
rs3735453	AA	42	3.21±2.62	2.56±1.34*	2.08±1.01*	2.36±1.12*	2.33±1.49*	2.35±1.63*	2.48±1.56*
(*ZSCAN25*)	AG	25	1.91±1.15	1.35±0.77	1.18±0.57	1.35±0.58	1.21±0.57	1.24±0.56	1.46±0.70
	GG	8	1.11±0.49	0.74±0.42	0.73±0.25	0.83±0.34	0.78±0.26	0.78±0.28	0.84±0.30
rs15524	TT	40	3.24±2.68	2.59±1.37*	2.13±1.04*	2.06±1.05*	2.26±1.41*	2.31±1.61*	2.43±1.54*
(*CYP3A5*)	CT	26	1.99±1.12	1.48±0.93	1.19±0.49	1.35±0.89	1.40±1.06	1.39±0.89	1.64±0.99
	CC	9	1.08±0.47	0.74±0.39	0.76±0.25	0.86±0.40	0.80±0.25	0.78±0.26	0.83±0.28
rs776746	GG	42	3.19±2.62	2.53±1.30*	2.09±1.02*	2.04±1.04*	2.26±1.38*	2.31±1.58*	2.47±1.54*
(*CYP3A5*)	AG	25	1.94±1.20	1.40±0.94	1.16±0.49	1.32±0.91	1.32±1.05	1.30±0.84	1.49±0.81
	AA	8	1.11±0.49	0.74±0.42	0.73±0.25	0.85±0.42	0.78±0.26	0.78±0.28	0.84±0.30
rs10211	AA	43	3.21±2.59	2.58±1.33*	2.11±1.02*	2.11±0.13*	2.35±1.48*	2.37±1.61*	2.51±1.56*
(*CYP3A7*)	GA	24	1.84±1.12	1.26±0.65	1.09±0.35	1.15±0.39	1.13±0.41	1.15±0.34	1.36±0.51
	GG	8	1.11±0.49	0.74±0.42	0.73±0.25	0.85±0.42	0.78±0.26	0.78±0.28	0.84±0.30
rs12360	TT	43	3.21±2.59	2.58±1.33*	2.11±1.02*	2.11±0.13*	2.35±1.48*	2.37±1.61*	2.51±1.56*
(*CYP3A7*)	TC	24	1.84±1.12	1.26±0.65	1.09±0.35	1.15±0.39	1.13±0.41	1.15±0.34	1.36±0.51
	CC	8	1.11±0.49	0.74±0.42	0.73±0.25	0.85±0.42	0.78±0.26	0.78±0.28	0.84±0.30
rs2257401	GG	38	3.47±2.65*	2.77±1.30*	2.23±1.02*	2.23±1.16*	2.48±1.52*	2.45±1.70*	2.63±1.60*
(*CYP3A7*)	CG	28	1.76±1.07	1.25±0.61	1.11±0.34	1.17±0.37	1.18±0.40	1.26±0.44	1.42±0.58

	CC	9	1.15±0.48	0.78±0.40	0.78±0.29	0.87±0.40	0.79±0.24	0.81±0.28	0.86±0.29
rs12333983 (*CYP3A4*)	TT	43	3.17±2.60	2.57±1.32*	2.12±1.02*	2.08±1.11*	2.35±1.48*	2.38±1.61*	2.52±1.55*
	AT	25	1.92±1.15	1.27±0.73	1.07±0.34	1.20±0.60	1.11±0.39	1.12±0.34	1.32±0.50
	AA	7	1.02±0.45	0.75±0.45	0.73±0.26	0.84±0.46	0.77±0.28	0.80±0.29	0.84±0.33
rs2242480 (*CYP3A4*)	CC	47	3.14±2.53	2.46±1.30*	2.02±1.02*	1.98±1.11*	2.21±1.48*	2.24±1.60*	2.42±1.53*
	CT	25	1.66±0.91	1.19±0.82	1.02±0.35	1.21±0.63	1.12±0.46	1.10±0.41	1.24±0.50
	TT	3	0.78±0.20	0.58±0.18	0.69±0.20	0.69±0.27	0.79±0.38	0.89±0.42	0.89±0.41
rs3814055 (*NR1I2*)	CC	45	2.79±2.59	2.10±1.34*	1.65±1.00	1.72±1.14	1.90±1.50	1.96±1.64	2.14±1.58*
	CT	25	2.19±1.49	1.81±1.31	1.65±1.00	1.59±0.90	1.59±1.02	1.58±0.99	1.74±1.02
	TT	5	2.20±1.21	1.52±0.81	1.52±0.68	1.61±0.65	1.81±0.70	1.65±0.59	1.56±0.68

Data are presented as mean with standard deviation. **P* value < 0.05 by ANOVA of linear regression adjusted based on the FDR correction

Supplementary Table S5 Diplotype frequencies of *CYP3A5* rs15524 and rs776746, *CYP3A7* rs10211 and rs2257401, and *CYP3A4* rs12333983 in the discovery set (N=75)

Group	Diplotype	N	Frequency (%)
TGAGT - TGAGT	TGAGT-TGAGT	34	45.3
TGAGT - CAGCA	TGAGT-CGAGT	21	28.0
CAGCA - CAGCA	AGCA-AGCA	7	9.3
Other	TGAGT-TGACT	5	6.7
	TGAGT-CGAGT	2	2.7
	TGAGT-TAAGT	1	1.3
	TGAGT-TGAGA	1	1.3
	TGAGT-CAGCT	1	1.3
	TGACT-CAGCA	1	1.3
	CAGCA-CAGCT	1	1.3
	CGAGT-CAGCA	1	1.3

Supplementary Table S6 Tacrolimus trough levels over time according to haplotypes of *CYP3A5* rs776746, *CYP3A7* rs10211 and rs2257401, and *CYP3A4* rs123339839 in the discovery set (N=75)

Diplotype	N	Daily dose adjusted tacrolimus trough levels (ng/mL per mg/day)						
		Day 3	Day 7	Day 14	Month 1	Month 3	Month 6	Month 12
TGAGT - TGAGT	34	3.50±2.79*	2.73±1.32*	2.29±1.05*	2.14±1.06*	2.55±1.12*	2.42±1.47*	2.43±1.71*
Others	34	1.92±1.11	1.44±0.92	1.18±0.45	1.37±0.88	1.39±0.61	1.37±0.94	1.40±0.82
CAGCA - CAGCA	7	1.02±0.45*	0.75±0.45*	0.73±0.26*	0.84±0.46*	0.77±0.28*	0.80±0.29*	0.84±0.33*

Data are presented as mean with standard deviation. **P* value<0 by ANOVA of linear regression.

Supplementary Table S7 Blood biochemistry data of patients at different time points after transplantation

Variables	Day 3	Day 7	Day14	Month 1	Month 3	Month 6	Month 12
Discovery group							
Hematocrit (%)	31.6±4.3	32.3±4.5	32.7±4.0	35.8±4.3	37.2±4.4	39.9±4.0	41.4±4.4
Total cholesterol (mg/dL)	155.2±33.4	164.9±33.9	168.0±25.4	187.4±36.3	174.4±37.5	177.7±32.2	184.1±30.1
Serum albumin (g/dL)	3.5±0.4	3.5±0.4	3.6±0.4	4.1±0.4	4.3±0.3	4.4±0.3	4.4±0.3
Total bilirubin (mg/dL)	0.4±0.1	0.6±0.2	0.6±0.2	0.6±0.2	0.6±0.2	0.6±0.2	0.7±0.3
Serum creatinine (mg/dL)	2.3±1.9	1.4±0.8	1.3±0.6	1.3±0.5	1.3±0.4	1.3±0.4	1.3±0.4
Alanine transaminase (U/L)	11.7±10.4	32.8±40.9	34.3±39.9	26.0±20.4	19.6±13.7	17.8±10.7	20.6±16.0
Replication group							
Hematocrit (%)	31.1±3.9	32.2±4.3	32.0±3.7	35.2±3.9	37.6±3.9	40.3±4.4	41.2±6.7
Total cholesterol (mg/dL)	152.8±34.3	161.5±29.6	170.8±37.5	187.1±42.0	179.2±32.4	179.3±34.0	174.6±38.4
Serum albumin (g/dL)	3.4±0.3	3.5±0.4	3.6±0.3	4.1±0.4	4.3±0.3	4.3±0.3	4.3±0.3
Total bilirubin (mg/dL)	0.4±0.2	0.6±0.2	0.6±0.3	0.6±.3	0.6±0.3	0.7±0.3	0.8±0.4
Serum creatinine (mg/dL)	2.2±1.7	1.6±1.3	1.4±0.8	1.3±0.4	1.3±0.3	1.3±0.3	1.2±0.3
Alanine transaminase (U/L)	11.6±7.7	35.2±33.9	33.8±40.2	25.2±17.3	17.8±8.8	20.1±13.6	19.1±10.6

Data are presented as mean with standard deviation.

Supplementary Table S8 Allele frequencies of *CYP3A4*, *CYP3A4*, and *CYP3A7* in the replication set (N=72).

Gene	Variants	Genotype	Frequency[a], N (%)	MAF (%)	HWE *P* value
CYP3A5	rs15524	CC	6 (8.3)	29.2	0.865
		CT	30 (41.7)		
		TT	36 (50.0)		
	rs776746	AA	6 (8.3)	27.8	0.939
		AG	28 (38.9)		
		GG	38 (52.8)		
CYP3A7	rs10211	GG	6 (8.3)	27.8	0.939
		AG	28 (38.9)		
		AA	38 (52.8)		
	rs12360	CC	6 (8.3)	27.8	0.939
		CT	28 (38.9)		
		TT	38 (52.8)		
	rs2257401	CC	6 (8.3)	29.9	0.944
		CG	31 (44.4)		
		GG	35 (47.2)		
CYP3A4	rs12333983	AA	5 (6.9)	29.2	0.674
		AT	32 (44.4)		
		TT	35 (48.6)		
	rs2242480	TT	5 (6.9)	27.1	0.916
		CT	29 (40.3)		
		CC	38 (52.8)		

[a]Frequency is given as the number of patients, with the percentage of patient cohort given in parenthesis. MAF, minor allele frequency; HWE, Hardy–Weinberg equilibrium

Supplementary Table S9 Tacrolimus trough levels over time according to genotypes in the replication set (N=72)

SNP (Gene)	Allele	N	Daily dose adjusted tacrolimus trough levels (ng/mL per mg/day)						
			Day 3	Day 7	Day 14	Month 1	Month 3	Month 6	Month 12
rs15524	TT	35	3.49±1.88	2.72±1.57	2.31±1.20	2.59±1.07	2.26±0.86	2.20±0.77	2.39±0.87
(*CYP3A5*)	CT	29	1.58±1.19	1.24±0.73	1.07±0.56	1.17±0.58	1.18±0.74	1.15±0.76	1.32±1.02
	CC	6	0.74±0.34	0.60±0.25	0.67±0.22	0.68±0.22	0.76±0.35	0.62±0.16	0.75±0.17
rs776746	GG	38	3.38±1.89*	2.68±1.54*	2.30±1.20*	2.56±1.07*	2.28±0.95*	2.22±0.89*	2.45±1.08*
(*CYP3A5*)	AG	28	1.57±1.19	1.16±0.68	0.98±0.41	1.10±0.47	1.06±0.38	1.02±0.31	1.14±0.39
	AA	6	0.74±0.34	0.60±0.25	0.67±0.22	0.68±0.22	0.76±0.35	0.62±0.16	0.75±0.17
rs10211	AA	37	3.38±1.89	2.68±1.54	2.30±1.20	2.56±1.07	2.28±0.95	2.22±0.89	2.45±1.08
(*CYP3A7*)	GA	28	1.57±1.19	1.16±0.68	0.98±0.41	1.10±0.47	1.06±0.38	1.02±0.31	1.14±0.39
	GG	5	0.77±0.38	0.53±0.21	0.67±0.25	0.67±0.25	0.73±0.39	0.63±0.18	0.81±0.11
rs12360	TT	38	3.38±1.89	2.68±1.54	2.30±1.20	2.56±1.07	2.28±0.95	2.22±0.89	2.45±1.08
(*CYP3A7*)	TC	28	1.57±1.19	1.16±0.68	0.98±0.41	1.10±0.47	1.06±0.38	1.02±0.31	1.14±0.39
	CC	4	0.85±0.36	0.71±0.17	0.72±0.19	0.76±0.18	0.84±0.31	0.68±0.11	0.75±0.19
rs2257401	GG	35	3.34±1.91	2.63±1.57	2.20±1.23	2.48±1.09	2.20±0.98	2.15±0.92	2.32±1.09
(*CYP3A7*)	CG	31	1.78±1.40	1.36±0.95	1.21±0.78	1.33±0.82	1.27±0.69	1.23±0.64	1.43±0.84
	CC	6	0.74±0.34	0.60±0.25	0.67±0.22	0.68±0.22	0.76±0.35	0.62±0.16	0.75±0.17
rs12333983	TT	36	3.25±2.01	2.62±1.63	2.22±1.27	2.48±1.17	2.20±1.02	2.16±0.97	2.31±1.16
(*CYP3A4*)	AT	32	1.75±1.31	1.29±0.79	1.11±0.61	1.24±0.64	1.21±0.59	1.14±0.50	1.35±0.71

	AA	4	0.87±0.35	0.61±0.13	0.75±0.18	0.76±0.18	0.86±0.32	0.70±0.09	0.86±0.04
rs2242480	CC	38	3.23±1.95	2.56±1.62	2.12±1.27	2.39±1.18	2.07±0.93	2.06±0.84	2.20±0.91
(*CYP3A4*)	CT	29	1.67±1.34	1.19±0.73	1.10±0.58	1.21±0.62	1.20±0.63	1.13±0.55	1.30±0.76
	TT	3	0.83±0.52	0.54±0.29	0.68±0.32	0.70±0.34	0.77±0.47	0.62±0.25	0.78±0.14

Data are presented as mean with standard deviation. *P value < 0.05 by ANOVA of linear regression adjusted based on the FDR correction

Supplementary Table S10. The oligonucleotide primers used for Sanger sequencing

Gene	Variants	RefSeq id	Location	Primer set
CYP3A7	rs2257401	NG_007983.1	g.31137C>G	Forward: ATGATACTGTGC<br>Reverse: CTGTGACTGGCTATAG

RefSeq, reference sequences

Supplementary Table S11. The oligonucleotide primers and melting temperature (T_m) used for SNaPShot assays

Gene	Variant	Tm (ºC)	Primer set
CYP3A7	rs2257401	55	Forward: CTTGACATGGTGGTGAATG<br>Reverse: TAAGTTGCTGGGACTGTGA<br>SNP primer: TCATSATGACCCAAAGTACTGGA
CYP3A7	rs12360	55	Forward: TGGAGCCTGATTTCCCTAAG<br>Reverse: AATGTGCAGGAAACATCCAA<br>SNP primer: GGTGTTCTGGGRCACAGCTTTCTT
CYP3A4	rs12333983	55	Forward: GTGGGGCCTTTGTCAGAAC<br>Reverse: GATCGGGGCACTAAGTGTGT<br>SNP primer: GAAATTAGATTGGAATGGATGTA
CYP3A4	rs2242480	60	Forward: CCAGCAGAAACTGCAGG<br>Reverse: GAGTCAGTGAAAGAATCAGTGATT<br>SNP primer: TACCCAATAAGGTGAGTGGATG
CYP3A4	rs4646437	60	Forward: TTTTATCCTTCAAAAGATGCACA<br>Reverse: TCTTTCAGGCCAGTGGCT<br>SNP primer: GGCAGGTCTATGCATAAGGAGCACC
CYP3A5	rs776746	60	Forward: TTATGGAGAGTGGCATAGGA<br>Reverse: GCTGATTAAACTTCACTAGCC<br>SNP primer:<br>CTCTTTAAAGAGMTCTTTTGTCTTTCA
CYP3A5	rs15524	55	Forward: TGGATGGTGAGTGCTTTTA<br>Reverse: AGACTCTGGGAGAGCTCAA<br>SNP primer: GGAGAATGAGTTATTCTAAGGA
ABCB1	rs1045642	55	Forward: TCTTGTTTTCAGCTGCTTG<br>Reverse: AACCCAAACAGGAAGTGTG<br>SNP primer: GGTGGTGWCACAGGAAGAGAT
ABCB1	rs2032582	55	Forward: CAAATCTTGGGACAGGAAT<br>Reverse: GGCCTGAAAACTGAAAAAG<br>SNP primer:<br>GAAAGATAAGAAAGAACTAGAAGGT

NR1I2	rs2276707	55	Forward: GTGAGGGGAGAGATGAGAG
			Reverse: CTGCAGCTTCTTCAGCAT
			SNP primer: GCTTGCTGAGAAGCTGCCCCTCCAT
SLCO1B3	rs4149117	55	Forward: TTGAGGGAAGGTACAATGTC
			Reverse: GGTGAAGTTGTGAAGCCTTA
			SNP primer: TGGGAAMTGGAAGTATTTTGACA
SLCO1B3	rs7311358	55	Forward: CTGGATCTACCCTTGAAAT
			Reverse: GATTATTAATGGATTTATTTCCTAC
			SNP primer: GATCTACATATCCAATATCCACGTA
SUMO4	rs237024	55	Forward: AGATCAGATTCCGATTTGGT
			Reverse: CAATTGAACAAGGTATGTGAGA
			SNP primer: TACCAGYTACTTCATGTATAATAAA

SNP, single nucleotide polymorphism

Supplementary Table S12. The oligonucleotide primers for the SNPtype assay

Gene	Variant	Primers	Oligonucleotide sequence
ABCB1	rs1128503	Allele specific primers	TCTGCACCTTCAGGTTCAGG
			CTCTGCACCTTCAGGTTCAGA
		Locus specific primers	GCCTTGAAGTTTTTTTCTCACTCGTCC
		Specific target primers	CCACCGTCTGCCCACT
ABCC2	rs2273697	Allele specific primers	ACATCAGGTTCACTGTTTCTCCAAT
			CATCAGGTTCACTGTTTCTCCAAC
		Locus specific primers	CCAACTTGGCCAGGAAGGAGTA
		Specific target primers	CCATGAGCTTCTGGGCATC
ABCC2	rs2804402	Allele specific primers	ACTCCAGGCTTCAACAATCCTC
			ACTCCAGGCTTCAACAATCCTT
		Locus specific primers	GCTCATGCCTGCAATCCCAG
		Specific target primers	GCCCAGGCTGGTCTCAAA
ABCC2	rs3740066	Allele specific primers	ACCTACCTTCTCCATGCTACCA
			CCTACCTTCTCCATGCTACCG
		Locus specific primers	CCTGAGCTGGATCTGGTCCT
		Specific target primers	AGGCCTTCCTTCACTCCA
ABCG2	rs2231142	Allele specific primers	CCGAAGAGCTGCTGAGAACTT
			CCGAAGAGCTGCTGAGAACTG
		Locus specific primers	TGATGGGCACTCTGACGGT
		Specific target primers	CATGATTCGTCATAGTTGTTGCAAG
CYP3A5	rs15524	Allele specific primers	CCTAAGTGGAGAATGAGTTATTCTAAGGAC
			CCTAAGTGGAGAATGAGTTATTCTAAGGAT
		Locus specific primers	GGCACAGCTTTCTTGAAGACCA
		Specific target primers	GTGGATTCAAGAGATGGAACCC
NR1I2	rs3814055	Allele specific primers	TCATTTTTTGGCAATCCCAGGTTC
			TCATTTTTTGGCAATCCCAGGTTT
		Locus specific primers	GGAGACCACGATTGAGCAAACA
		Specific target primers	ATGTTCACCTGAAGACAACTGTG
NR1I2	rs6785049	Allele specific primers	CCATCCTCCCTCTTCCTCTCA
			CATCCTCCCTCTTCCTCTCG

		Locus specific primers	GCACCAGCAGCCATCCCATA
		Specific target primers	AGTCATCCTCAGGGAAAGGAG
POR	rs1057868	Allele specific primers	CGCCGTTCTCCCCGG
			CGCCGTTCTCCCCGA
		Locus specific primers	GGCCGCATCAACAAGGG
		Specific target primers	GAACATGGGCACCAGCG
POR	rs2868177	Allele specific primers	GACTTTGGATCCTACACAGTGAGT
			ACTTTGGATCCTACACAGTGAGC
		Locus specific primers	GTCTCCCTGCCTCAGCCT
		Specific target primers	AGCCCTGGATCCTGTGG
PPARA	rs4823613	Allele specific primers	ATCCATTAAAAGCCACATTTAACCCAA
			ATCCATTAAAAGCCACATTTAACCCAG
		Locus specific primers	TGCCCGGCCTGCTTT-TAATTT
		Specific target primers	TGCATACTAGCCTTGTGGTCTA
PPARD	rs2267668	Allele specific primers	TGAGTTTGAGCTGTCGGTAAAATATCTA
			GAGTTTGAGCTGTCGGTAAAATATCTG
		Locus specific primers	GCATCTGTGGGACCC-CAAC
		Specific target primers	AAAATGATGGGTTCTGTTTGGATAGA
SUMO4	rs237025	Allele specific primers	CAAATCGGAATCTGATCTGCTTCAC
			CAAATCGGAATCTGATCTGCTTCAT
		Locus specific primers	TGTGAACCACGGGGA-TTGTCA
		Specific target primers	TTGTCTGTTCCACTGATTGGTTG
